# Cost-of-illness for non-underweight binge-eating disorders

**DOI:** 10.1007/s40519-021-01277-3

**Published:** 2021-07-30

**Authors:** Paul E. Jenkins

**Affiliations:** School of Psychology and Clinical Language Sciences, University of Reading, Reading, RG6 6ES UK

**Keywords:** Healthcare costs, Healthcare utilisation, Binge eating, Impairment

## Abstract

**Purpose:**

This study examined economic costs associated with untreated eating disorders (EDs) characterised by regular binge eating in the absence of low weight. Both direct and indirect costs were assessed, reporting a limited societal perspective of economic impact as some costs were not included.

**Methods:**

One hundred and twenty six adults seeking treatment for recurrent binge eating were asked to report impairment associated with an ED. Costs were calculated using 2017 prices, including an examination of variables associated with costs.

**Results:**

Estimated societal costs for the year preceding assessment were £3268.47 (€3758.54) per person. In multivariate analyses, no reliable baseline associates of cost were identified.

**Conclusion:**

The economic burden of EDs characterised by regular binge eating is significant, and underscores the need for efficacious and cost-effective treatments. Individuals with binge-eating disorders report work impairment and healthcare use that may cost the United Kingdom economy upwards of £3.5 billion (€4bn) per annum. Further studies should consider academic impairment and the economic impact of EDs on families.

**Level of evidence:**

III: evidence obtained from well-designed cohort or case–control analytic studies.

**Supplementary Information:**

The online version contains supplementary material available at 10.1007/s40519-021-01277-3.

## Introduction

Attention paid to the economic burden of eating disorders (EDs) has been increasing, particularly over the last two decades [1]. Given the significant morbidity associated with EDs, attempts have been made to estimate cost-of-illness, covering both ‘direct’ and ‘indirect’ costs, often referred to as taking a societal perspective [2]. Direct costs encompass healthcare and non-healthcare costs involved in the treatment and care of an illness and indirect costs provide an estimate of economic resources lost because of impaired productivity (e.g., employment-related). In addition, ‘psychosocial’ costs assess the (often intangible) impact of an illness on quality of life or well-being and are perhaps the least well-understood contributor to economic burdens. The societal perspective, which typically includes an assessment of all relevant costs, informs policies and evaluations aimed at maximising welfare gains, considering impacts on both healthcare and wider society [3]. Thus, although costs can be usefully categorised in several ways (e.g., resources for medical and non-medical care), economic reporting within the societal perspective proceeds regardless of who bears this cost [3].

Reviews of cost-of-illness studies in EDs have underlined heterogeneity in samples and methods used, resulting in wide variation in estimated costs [1, 4] (see also [5]). A large proportion of studies originate from North America and many obtain cost information from health insurance databases (see also [6]), which can be affected by sampling bias as well as variable insurance coverage across regions [4]. There has been limited consideration of wider costs, and studies have underestimated economic impact [1], rarely covering factors such as work absence [4]. Research taking a societal perspective is often lacking [1], meaning that the true cost-of-illness may be underestimated and costs relevant to the wider impact of EDs overlooked.

Comparatively little is known about the economic impact of regular binge eating, with a shortage of published studies from the United Kingdom (UK) (and Europe), where accessibility of healthcare is underpinned by universal coverage or state-funded services, often ‘free at the point of delivery’. Individuals with regular binge eating who are not underweight represent the majority of those with EDs presenting for outpatient treatment [7] and are rarely afforded consideration as a distinct group in cost-of-illness studies, although might be partially captured in studies including different types of EDs (e.g., [6, 8]). Existing work has tended to focus on one particular illness rather than considering a range of ED presentations [1, 6, 8–11], with studies of anorexia nervosa particularly common.

Healthcare use for non-underweight individuals who report regular binge eating is higher than that for individuals without EDs [10, 12, 13], even when accounting for comorbidities [6, 14]. When examining economic impact, annual healthcare costs for bulimia nervosa (BN) range from €888 to €18,823, and €1762 to €2902 for individuals with binge-eating disorder (BED) [15], with societal costs seldom reported [16] (but see [5, 17, 18]). Data on individuals with subthreshold EDs and those with a diagnosis of other specified feeding and eating disorder (OSFED) are notably lacking given the estimated prevalence of these syndromes and the associated impairment and cost [19].

Although some studies have examined the influence of demographic factors, such as age and gender (e.g., [6, 11]), few have explored whether common ED symptoms, such as binge eating, are associated with costs, which can help inform both healthcare and research priorities. Looking at a sample of women seeking treatment for regular binge eating, Dickerson et al. [20] found that baseline binge eating frequency was not associated with costs, although age was positively related to both medication costs and total healthcare costs. Higher body mass index (BMI) was positively associated with medication costs (see also [5]).

A 2015 report commissioned by a UK ED charity [21] suggested an annual cost of between £2.6 billion and £3.1bn to sufferers and carers, with costs to the National Health Service (NHS) of between £3.9bn and £4.6bn. A report of societal costs to the US in 2018–2019 [5] estimated costs exceeding $64bn (equivalent to $11,808 [~ €9784] for each person with an ED). Similar estimates have been made in other countries (e.g., [16, 22]) and outline the significant financial burden of EDs although further work is required to explore and triangulate these findings [22].

The current study aims to estimate societal costs in a group of non-underweight individuals referred for specialist outpatient treatment for regular binge eating, using a prevalence-based, ‘bottom–up’ approach (i.e., obtaining cost data directly from patients via self-report). Additionally, the study will look at associations between costs, demographic factors, and binge eating, as well as exploring diagnostic differences (e.g., [13, 14, 23]).

## Materials and methods

### Participants

Participants were 126 adults referred to one of three specialist ED services in the UK, covering a population of around 1.3 million adults across Buckinghamshire, Oxfordshire, and Wiltshire. (A recent report [24] compiled a directory of 56 similar services across England.) Individuals who, at assessment with the service, met criteria for a diagnosis of BN, BED, or OSFED participated in a randomised controlled trial of guided self-help with few exclusion criteria (see [25]), and data from these individuals were included in the current study (see Table 1). Conduct of the trial was approved by an ethics review board (details are reported elsewhere [26]) and the study was performed in accordance with the ethical standards as laid down in the 1964 Declaration of Helsinki and its later amendments.

**Table 1:** Demographic and clinical characteristics of individuals with non-underweight binge-eating disorders

Variable	Value
Gender, female:male^a^	118:8
Age, years: mean (SD)	30.20 (10.24)
Duration of illness, years: mean (SD)^b^	11.44 (9.87)
Body mass index, kg/m^2^: mean (SD)^c^	27.42 (8.74)
Ethnicity	
White—British White—other Mixed Other Not stated	104 (82.5%) 14 (11.1%) 2 (1.6%) 2 (1.6%) 4 (3.2%)
Eating disorder diagnosis	
Bulimia nervosa: *n* (%)	76 (60.3)
Binge-eating disorder: *n* (%)	27 (21.4)
Other specified feeding and eating disorder: *n* (%)	23 (18.3)
Employment status	
Employed: *n* (%)	80 (63.5)
Unemployed: *n* (%)	7 (5.6)
Full-time student: *n* (%)	34 (27.0)
Other: *n* (%)	5 (4.0)

*N* = 126, except where indicated

^a^All identified with either male or female gender

^b^= 113

^c^*N* = 123

### Healthcare utilisation

A questionnaire was designed for this study, completed retrospectively by participants (see [25]). Although self-report methods of estimating economic impact can be challenging, it is recommended that they consider: a recall time frame of 6 months or less; resource utilisation frequency; and type of utilisation [27, 28]. The questionnaire asked participants about healthcare utilisation over the previous 3 months and to estimate of the amount of time lost from work or education (i.e., absenteeism) and reduced productivity (i.e., presenteeism). Information was requested regarding medical investigations and out-of-pocket expenses for attending appointments, specifically travel costs. Centralised NHS records were not accessed, although information about medication use was obtained from patient records.

The suggestions of Jo [2] were used as to guide economic analyses, although shortcomings in the methods meant that some costs (e.g., regarding carers, social care, foregone leisure activities) were not gathered and so the estimate is referred to as being from a ‘limited societal’ perspective [3]. As the questionnaire covered a 3-month period, annual figures are provided, including an estimate of the cost to society through multiplying annual costs per patient by the prevalence of binge eating problems (1.6%) in a sample of the population of England [29]. Cost data are reported in pound sterling (GBP), with summaries converted to Euros for comparative purposes (using a rate of £1:€1.15 [May 6 2021]).

### Estimating unit costs

Total costs were calculated by taking the average usage for the sample and multiplying this by the unit cost. Prices were obtained through either (1) the Unit Costs of Health and Social Care [30] or (2) a policy paper regarding NHS reference costs 2013–2014 [31]; the latter was adjusted to 2017 prices using the Consumer Price Index rate (details from [32]). Cost details are provided in Online Resource 1 covering several types of appointment. Where participants stated that they used a service but did not specify the number of visits, a value of “1” was given, which is likely to underestimate use.

In the UK, individuals are entitled to healthcare which is free at the point of access. For example, appointments with a primary care physician or attendance at Accident and Emergency departments will generally be free of charge to the patient. Some interventions (e.g., medications) do incur a charge although some individuals (e.g., women who are pregnant or who have had a baby in the last year) are entitled to free prescriptions.

### Work and study impairment

Absenteeism costs were estimated from responses to the question: “How many full days have you lost from work due to eating disorder symptoms or concerns in the last three months?” As presenteeism, defined as reduced productivity whilst at work, is more complex to estimate, this was based on a study of individuals with BED [33] reporting around 30% of time lost due to impaired productivity. Thus, if a participant reported 10 days of reduced productivity (“How many full days of reduced productivity have you experienced while at work due to eating disorder symptoms or concerns in the last three months?”), this was ‘costed’ as the equivalent of three days of lost work. (A ratio of around 1:2.1 was estimated in a study of depression [34]). Study impairment is presented as number of full days lost and days affected by reduced productivity regarding education; no economic estimates are attributed to this.

To calculate costs, the Human Capital Method was used (see [2, 35]). This involved taking the number of days missed in the last three months, multiplying this by the equivalent mean wage (£16.63 per h,^1^ based on that of a female in her early 30 s [36]) and extrapolating for 1 year. Some individuals provided data for productivity costs but not direct costs and were excluded from primary analyses but included in a sensitivity analysis, with missing data (typically regarding travel) costed as 0 (see Online Resource 2). A further sensitivity analysis (Online Resource 3) used different prevalence rates for men (0.5%) and women (2.5%) [29] and a third (Online Resource 4) adjusted wage estimates by gender. A fourth sensitivity analysis was based on medication use and assumed a single unit cost for medication (Online Resource 5).

Binge eating frequency was taken from the Eating Disorder Examination Questionnaire (EDE-Q; [37]), collected at the same time as cost data. Therefore, the timeframe of cost estimates was longer than that assessed by the EDE-Q (28 days), meaning that temporal precedence cannot be determined.

### Statistical analyses

Kolmogorov–Smirnov tests for the normality of variable distribution were significant for costs (*p*s < 0.01). The Kruskal–Wallis *H* Test was used to look at group differences and the generalised linear model (GLM) with gamma probability distribution and log link function was used to explore associations with binge eating when controlling for covariates (age, BMI, gender). Only main effects were considered given small sample sizes and issues with interpreting interaction terms in some GLMs (see [38]). Estimates of societal costs are provided alongside bootstrap-based 95% confidence intervals (CIs) [4].

## Results

Healthcare use and associated costs are provided in Table 2. Inpatient costs were zero as no patients were admitted in the three months prior to assessment. Most participants had contacted a primary care physician and over half had undergone investigations such as blood tests. Just over one-quarter had taken psychotropic medication.

**Table 2:** Summary of healthcare resource use and estimated costs associated with binge-eating disorders (*N* = 126)

Domain	% with any use	Total no. of visits (last 3 months)	Mean no. of contacts for users (last 3 months)	Unit cost	Cost per person per year	Component as % of total annual healthcare cost
Primary care physician	74/86 (86.0%)	NK	1	£38.00	£130.80	16.03%
Other healthcare professional	31/84 (36.9%)	61	0.73	£39.00	£113.88	13.96%
Accident and emergency department	3/84 (3.6%)	13	0.15	£106.42	£65.88	8.08%
Medication						
Antidepressants	33/126 (26.2%)	–	–	£15.66	£16.40	2.01%
Other	3/126 (2.4%)	–	–	£24.33	£2.32	0.28%
Additional costs (e.g., dispensing)	36/126 (28.6%)	–	–	£385.20	£440.24	53.97%
Medical investigations^a^						
Blood tests	45/85 (52.9%)	47	0.55	£8.41	£18.60	2.28%
Cardiac investigations	7/85 (8.1%)	7	0.08	£40.97	£13.48	1.65%
Bone density	3/85 (3.5%)	3	0.04	£72.32	£10.20	1.25%
Other	4/85 (4.7%)	4	0.05	£21.00	£3.96	0.49%
Subtotal	–	–	–	–	(£46.24)	(5.67%)

^a^Participants could report > 1 investigation and could specify the frequency (e.g., “two blood tests”). One participant reported having received an investigation but did not disclose its nature (so was costed as zero)

*NK* not known

Work absenteeism was reported by 40.0% of participants and 60.0% reported missing days of study/education (see Table 3). The majority of participants reported at least some work (*n* = 24; 84.5%) or study (*n* = 19; 82.6%) presenteeism. Eleven individuals gave information regarding both work and study.

**Table 3:** Lost work and study productivity estimates (mean [SD]) for non-underweight individuals with binge eating

Domain	*N*^a^	Days (last 3 months)	Annual days	Estimated cost per annum
Work				
Absenteeism	77	1.88 (4.12)	7.52 (16.48)	£937.41 (2062.03)
Presenteeism	76	14.22 (19.66)	56.88 (78.64)	£2046.72 (2916.47)
Total	77	–	–	£2957.55 (3813.29)
Study				
Absenteeism	25	9.40 (18.60)	37.60 (74.40)	–
Presenteeism	23	28.70 (26.23)	114.78 (104.91)	–

^a^If data were missing from only one question, the cost is included in the total

### Out-of-pocket expenses

Costs were estimated based on travel to the Eating Disorders Service (EDS), with mileage costs based on £0.40 (€0.46) per mile travelled. Participants mentioned costs associated with missing work to attend appointments although this was sporadic and therefore not included.

Seventy-five individuals provided data on out-of-pocket expenses, of whom 28 (37.3%) cited no costs to themselves; for example, some used modes of transport from which running costs were not estimated (e.g., bicycle). As such, this is likely to be an underestimate of cost impact for patients, as costs to attend other appointments (e.g., blood tests) and possible medication charges were not included. Estimated out-of-pocket expenses for the previous 3 months varied from zero to £60, and were not extrapolated for the year as the majority related to attendance for assessment at the EDS.

### Costs by diagnosis

Costs by DSM-5 diagnosis [39] are presented in Table 4. Societal cost was estimated at £3268.47 (€3758.74) per person per annum across binge-eating disorders, with similar estimates and trends observed in sensitivity analyses (range for societal costs = £3169.87–£3316.14).

**Table 4:** Estimated costs of non-underweight binge-eating disorders by total sample and DSM-5 diagnosis, per individual per year

			Costs, mean (SD)		
Diagnosis	Healthcare use	Out-of-pocket	Productivity	Societal	Bootstrapped societal 95% CIs
Total sample	£474.29 (631.42)	£10.62 (12.76)	£2957.55 (3813.29)	£3268.47 (3985.74)	£2421.15–£4218.38
BN	£529.44 (784.78)	£8.95 (12.89)	£3246.88 (4172.74)	£3559.07 (4448.88)	£2336.23–£5078.95
BED	£376.40 (312.99)	£13.95 (10.81)	£1757.69 (2527.47)	£2144.08 (2475.77)	£1121.62–£3432.92
OSFED	£405.96 (192.83)	£12.33 (13.95)	£3366.00 (3671.25)	£3702.12 (3798.21)	£1861.18–£5699.20
*H* (*df* = 2)	1.456	4.204	1.849	1.596	N/A

All tests ns

### Correlates of costs

Overall regression models (including age, BMI, gender, binge eating frequency) were not significant for any costs (*p*s > 0.05). Costs by age group are presented in Online Resource 4.

## Discussion

The current study reports cost-of-illness data for adults referred for treatment of recurrent binge eating in the absence of significantly low body weight. Estimated costs to society were £3268.47 (€3758.74) per individual per annum, with similar findings obtained through sensitivity analyses. Tangible costs borne by UK society for individuals with such presentations equate to around £3.47bn per year; a sensitivity analysis (changing the estimate of prevalence) produced a figure of just over £3.1bn. These figures indicate the significant burden of non-underweight EDs; in 2010, for example, the UK societal cost of all anxiety disorders was estimated to be around €11.7bn and €1.6bn for epilepsy; [40]). Results should be considered indicative (rather than exact) and taken alongside those of other studies—such as appraisals obtained from health insurance databases [6, 13], prevalence-based reviews of existing data [5], and surveys [11, 17]—to estimate the overall economic impact of the full range of EDs.

Previous societal estimates have suggested that costs of EDs range between €10,000 and €14,000 per person per year and, around a decade ago, Mitchell et al. [23] estimated that individuals with EDs incur costs of around US$4000 (2005 prices) in the year preceding diagnosis, close to the current estimate (see also [13]). Costs of US$9541 [5] (~ €7946) per person per year have been attributed to BED, for example, although higher estimates have been noted (e.g., [16, 17]).

Around 8 million days of work may be lost through absenteeism in the UK per year, and an even greater number through presenteeism, due to binge eating problems. Findings are comparable to those regarding other psychiatric disorders (e.g., around nine annual days lost per worker with depression [34]), and economic reports in the US and elsewhere have estimated that 75% of the overall cost of EDs is attributable to productivity losses [5]. Given that the current cost estimate of presenteeism was based on previous work [33], which has also been the case in a recent US study [5], more work is needed to clarify productivity losses attributable to EDs. Assessment of academic impairment (see also [41]) suggested that around 40 days of study time per year are lost for each individual with a non-underweight binge-eating disorder. Study presenteeism was also high, although sample sizes were small. Given that academic impairment has rarely been considered in cost-of-illness studies [1], this area warrants further research.

Findings regarding multivariate models of costs were in line with some existing work (e.g., [11]) and no diagnostic differences were observed in cost estimates (see also [14, 23]). Although costs appeared to be lower in the oldest group (see also [5]), the lack of significant findings may have been influenced by sample size, with analyses not sufficiently powered to detect small correlations. In addition to larger samples, further research should consider other covariates, such as depression, given the contribution of comorbidity to higher cost estimates (e.g., [11, 20]).

Whilst the small sample size should be noted, men incurred lower societal costs than women (see Online Material 3) and, although the gender ratio is in line with a UK study conducted in primary care [42], men were likely underrepresented in the current study and the true cost impact of men with EDs may be notably underestimated, particularly given wage imbalances (e.g., see [5]). Much of the cost burden of healthcare noted in previous studies has been attributed to inpatient admission, with the average cost of a hospitalised individual approximately four times that of someone not admitted [1]. Although only a minority of individuals with binge-eating disorders receive hospital treatment [43], the current study assumed zero costs due to non-emergency admissions and additional reports of cost estimates for those commonly seen in outpatient clinics are needed.

The current estimates, whilst substantial and in line with some studies, are less than those presented in a recent report commissioned by a UK eating disorder charity [21] and those in other countries [5, 16, 22]. This is likely a result of several factors. First, only ‘tangible’ costs (e.g., healthcare, productivity) were included in the current study, with no assessment of the impact on wellbeing (see [5, 16]). Second, children with regular binge eating, individuals with anorexia nervosa, and costs to carers were not included.

### Strengths and limits

Several sensitivity analyses were reported and a further strength of the methodology was use of bootstrapping to account for data skew [4]. The study employed a timeframe of 3 months as a trade-off between accuracy of recall and coverage, which was then generalised to estimate one-year societal costs, in line with previous work. This was both a strength and a limitation, risking underestimation of annual costs, although few cost imputation methods are free from bias [28]. Although participants were asked to consider costs related to their ED, costs associated with comorbidity were not estimated (e.g., [6, 17]) and absence of a control group prevented direct comparisons with other samples (e.g., see [10]). The questionnaire assessing healthcare use (see [25]) has not undergone psychometric evaluation and use of centralised sources for cost estimation (e.g., see [44]) may complement ‘bottom-up’ studies such as the current one.

The human capital approach (see [35]) was used to estimate costs, based on the assumption that wages are a proxy measure of productivity losses. Alternative approaches adopt different perspectives and may produce different cost estimates (e.g., the friction cost approach; see [2, 35]). Several other assumptions were made across economic analyses, likely resulting in an underestimate of the true costs of binge eating. Although participants were invited to detail costs related to their illness not assessed by the questionnaire, responses were sporadic and omitted from the final analyses. Out-of-pocket expenses focused largely on transportation and may have overlooked costs directly related to binge eating (see [16]). Medication costs (which are complex to estimate [45]) were included as a Healthcare cost, thus likely underestimating costs to patients, although the economic burden is nonetheless captured in the societal estimate.

The societal estimate is considered ‘limited’ [3] as several costs were not captured, contributing to a probable underestimation of associated costs. Provision of informal care can be substantial [5] and there was no estimate of the economic impact of caring for someone with an ED, such as time spent attending joint appointments, and no assessment of expenditure on private treatment, which has been noted in previous UK studies [21]. Some questionnaire responses were missing information (e.g., number of visits to a healthcare professional), and the resulting assumptions also likely underestimated costs.

The current study is one of the first to estimate societal costs of EDs focusing on a sample characterised by regular binge eating in the absence of low weight. Although this population is at low risk of hospital admission [43], costs associated with both healthcare use and productivity losses were high, and comparable with previous estimates for similar disorders. Findings highlight the significant cost of EDs in individuals who are not underweight (those most commonly seen in clinical practice) and underscore the need for efficacious and cost-effective interventions. Paired with emerging data regarding traditional underinvestment in ED research [20], the findings should act as a call-to-arms to stimulate funding and direction of resources to reduce the burden of EDs.

### What is already known on this subject?

Existing cost estimates have highlighted the huge economic burden of EDs, costing society between €10,000 and €14,000 per person per year. However, several important limitations of previous work necessitate further studies in this area.

### What does this study add?

The current study looks at individuals reporting regular binge eating in the absence of low weight—perhaps the largest subgroup of EDs presenting to clinical services. The findings are in line with previous estimates (costs to society of €3758.74 per individual per annum) and suggest that greater investment in the treatment of binge eating is needed although few reliable correlates of cost were identified.

## Supplementary Information

Below is the link to the electronic supplementary material.

Supplementary file1 (DOCX 27 KB)

## Data Availability

The data that support the findings of this study are available from the author upon reasonable request. The data are not publicly available due to privacy or ethical restrictions (participants were not asked to provide consent for their data to be made publicly available).

## Footnotes

1 This equates to £623.63 per week. Median gross weekly earnings for full-time employees across the UK in 2017 was £550 [36].
