# Supplementary material for: Cost-of-illness for non-underweight binge-eating disorders
Source: Eat Weight Disord. 2021 Jul 30;27(4):1377–84. doi: 10.1007/s40519-021-01277-3 (PMC9079013; doi:10.1007/s40519-021-01277-3)
Supplement: Supplementary file 1 — Supplementary file1 (DOCX 27 KB) [file 40519_2021_1277_MOESM1_ESM.docx]

**Supplementary material**

#

**Cost-of-illness for non-underweight binge-eating disorders**

*Eating and Weight Disorders*

Paul E. Jenkins

University of Reading, UK

E-mail: pej106@gmail.com

Table S1

Calculation of costs for different categories of healthcare use

| Healthcare use | Cost (£) | Explanation |
| --- | --- | --- |
| Primary care physician visits | 38.00 | This was estimated for a consultation lasting 9.22 minutes [1] although data regarding number of visits was not recorded. |
| Other healthcare professional visits | 39.00 | This was estimated for an appointment with a mental health worker in the community [1]. |
| Accident & Emergency visit | 106.42 | This was estimated as a Category 1 Investigation with Category 1-2 Treatment [2] (£103.00 adjusted to £106.42 for 2017). |
| Medication   - Antidepressant - Antipsychotic - Dispensing cost - General practitioner prescribing costs | 5.22  8.11  0.91  4.28 | Details regarding psychotropic medications were taken from patients’ clinical notes. Information on other medications (e.g., analgesia, vitamin supplements) was not consistently available and therefore not reported. Costs were estimated from 2017 figures of the Prescription Cost Analysis, which provides details of all prescriptions dispensed in the community in England [3]. The cost of antidepressants was estimated at £5.22 per person per month; this is the Net Ingredient Cost (NIC), which does not include dispensing fees, container costs, or tax, and was based on 60mg fluoxetine hydrochloride capsules (e.g., [4]) and an “arbitrary assumption” that this represents one month’s worth of medication ([5], p. 188). To include an estimate of additional costs, we used data from Doble et al. [6]; specifically, an additional £0.91 per day was included for dispensing costs and £3.23 for general practitioner prescribing costs (2017 cost total = £4.28/day). There was no further information on dosage or frequency, which may be important for repeat prescribing, and also enables a more detailed analysis of costs [5]. Where participants reported use of other prescription drugs, these were estimated in a similar manner, using the guide of a low-dose (generic) antipsychotic at £8.11 NIC. Costs over a three-month period were therefore £15.66 for antidepressants, £24.33 for other drugs, and £385.20 for dispensing and prescribing. |
| Blood tests | 8.41 | Mehler and Andersen [7] recommend specific laboratory tests for individuals with EDs, including full blood count, electrolytes, and liver function tests. Costs were estimated through reference to an article by Akhtar and Chung [8], who provided data from one UK laboratory as follows: FBC £2.65; U&E £2.12; LFTs £2.78; magnesium £0.57. Given that other tests (e.g., phosphate, thyroid function tests) are often recommended but not included in this analysis, the overall cost is likely to be an underestimate. Estimated unit cost for participants reporting a blood test was £8.12, adjusted to £8.41. |
| Cardiac investigations | 40.97 | Cardiac investigations (e.g., electrocardiogram) were estimated from NHS Reference costs [9]: adjusted from £37.00 in 2011 to £40.97. |
| Dual energy X-ray absorptiometry | 72.32 | Estimated from NHS Reference costs [2]: adjusted from £70.00 to £72.32. |
| Other appointments | 21.00 | Where participants noted a different investigation not categorised above, cost was estimated based on 30 minutes of nurse working in GP practice time (£21.00; [1]). |

References

1. Curtis L, Burns A (2017) *Unit Costs of Health and Social Care 2017*. Canterbury: University of Kent. https://doi.org/10.22024/UniKent/01.02/65559
2. Department of Health and Social Care (2015). Reference costs 2013-14. <https://www.gov.uk/government/publications/nhs-reference-costs-2013-to-2014#history>. Accessed February 27 2021
3. NHS Digital (2018). Prescription Cost Analysis – England, 2017. <https://digital.nhs.uk/data-and-information/publications/statistical/prescription-cost-analysis/prescription-cost-analysis-england-2017>. Accessed July 11 2021
4. Crow S (2014) Treatment of Binge Eating Disorder. Curr Treat Options Psychiatry, 1:307–314. https://doi.org/10.1007/s40501-014-0023-4
5. Heslin M, Babalola O, Ibrahim F, Stringer D, Scott D, Patel A (2018) A comparison of different approaches for costing medication use in an economic evaluation. Value Health 21:185-192. https://doi.org/10.1016/j.jval.2017.02.001
6. Doble B, Payne R, Harshfield A, Wilson ECF (2017) Retrospective, multicohort analysis of the Clinical Practice Research Datalink (CPRD) to determine differences in the cost of medication wastage, dispensing fees and prescriber time of issuing either short (<60 days) or long (≥60 days) prescription lengths in primary care for common, chronic conditions in the UK. BMJ Open 7:e019382. https://doi.org/10.1136/bmjopen-2017-019382
7. Mehler PS, Andersen AE (2010) Eating Disorders: A Guide to Medical Care and Complications (2nd ed.). Johns Hopkins University Press, Baltimore, MD.
8. Akhtar W, Chung Y (2014) Saving the NHS one blood test at a time. BMJ Open Qual 2:u204012.w1749. https://doi.org/10.1136/bmjquality.u204012.w1749
9. National Institute for Health and Care Excellence (2016). Preoperative tests (update): Routine preoperative tests for elective surgery. Clinical guideline NG45. Author, London, UK.

Table S2

Sensitivity analysis (SA1) for non-underweight binge eating with cost data for productivity losses and missing data for indirect costs costed as zero

|  | Costs, mean (SD) | | | |
| --- | --- | --- | --- | --- |
| Diagnosis | Healthcare use | Out-of-pocket | Productivity | Societal |
| Total sample | £471.79 (642.14) | £9.20 (11.39) | £2957.75 (3813.69) | £3169.87 (3897.71) |
| BN | £527.52 (805.43) | £7.26 (10.34) | £3247.20 (4173.30) | £3600.42 (4396.76) |
| BED | £376.40 (312.99) | £12.31 (11.13) | £1757.69 (2527.47) | £2019.42 (2451.64) |
| OSFED | £405.96 (192.83) | £11.60 (13.84) | £3366.00 (3671.25) | £3789.44 (3650.12) |

Table S3

Sensitivity analysis (SA2) of costs of non-underweight binge eating adopting different prevalence estimates by gender

|  |  |  | Societal Costs | |
| --- | --- | --- | --- | --- |
| Gender | Population^a^ | Estimated prevalence^b^ | Individual, mean (SD) | Annual |
| Female | 33,645,348 | 2.5% | £3438.88 (4112.05) | £2,892,557,858.26 |
| Male | 32,790,202 | 0.5% | £1393.90 (1079.86) | £228,531,919.46 |
| Total | 66,435,550 | - | - | £3,121,089,777.72 |

*Note*. ^a^Office for National Statistics (2018). Dataset: Estimates of the population for the UK, England and Wales, Scotland and Northern Ireland: June 2018. https://www.ons.gov.uk/peoplepopulationandcommunity/populationandmigration/populationestimates/datasets/populationestimatesforukenglandandwalesscotlandandnorthernireland; ^b^McBride, O., McManus, S., Thompson, J., Palmer, R. L., & Brugha, T. (2013). Profiling disordered eating patterns and body mass index (BMI) in the English general population. Social Psychiatry and Psychiatric Epidemiology, 48, 783-793. https://doi.org/10.1007/s00127-012-0613-7

Table S4

Sensitivity analysis (SA3) assuming different wage estimates by gender (Women: £16.63ph, Men: £17.71ph).

|  | Costs, mean (SD) | |
| --- | --- | --- |
|  | Productivity Costs | Societal Costs |
| Total sample | £2962.34 (3812.26) | £3273.59 (3984.70) |

Table S5

Sensitivity analysis (SA4) assuming a single unit cost for medications, based on the net ingredient cost average for all medications listed in the UK Prescription Cost Analysis (£8.29), keeping prescription item costs the same.

|  | Costs, mean (SD) | | |
| --- | --- | --- | --- |
|  | N | Healthcare use | Societal |
| SA4 | 83 | £476.97 (632.45) | £3271.30 (3986.60) |

*Note*: This method is based on the recommendations of Patel et al. (2017); Patel, A., Heslin, M., & Babalola, O. (2017). Estimating medication costs for economic evaluation, in L. Curtis & A. Burns (eds) Unit Costs of Health & Social Care, Personal Social Services Research Unit. University of Kent: Canterbury.

Table S6

Estimated costs across age groups for non-underweight individuals reporting regular binge eating

|  | | Costs, mean (SD) | | | |
| --- | --- | --- | --- | --- | --- |
| Age group, years | N* | Healthcare use | Out-of-pocket | Productivity | Societal |
| 17 – 24 | 27 – 32 | £419.87 (318.56) | £9.36 (14.70) | £3295.11 (4531.92) | £3762.01 (4911.53) |
| 25 – 39 | 35 – 39 | £540.41 (866.91) | £9.04 (8.93) | £2948.52 (3342.50) | £3176.69 (3316.63) |
| 40+ | 10 – 12 | £404.54 (245.92) | £19.26 (15.37) | £2097.98 (3412.05) | £2257.12 (3489.93) |

*N varies for each cost, so minima and maxima are given
